# User-Dependent Usability and Feasibility of a Swallowing Training mHealth App for Older Adults: Mixed Methods Pilot Study

**DOI:** 10.2196/19585

**Published:** 2020-07-27

**Authors:** HyangHee Kim, Sang-Ho Lee, Nam-Bin Cho, Heecheon You, Teukgyu Choi, Jinwon Kim

**Affiliations:** 1 Graduate Program in Speech-Language Pathology Department and Research Institute of Rehabilitation Medicine Yonsei University College of Medicine Seoul Republic of Korea; 2 Graduate Program in Speech-Language Pathology Yonsei University Seoul Republic of Korea; 3 Department of Industrial and Management Engineering Pohang University of Science and Technology Pohang Republic of Korea; 4 Humanopia Pohang Republic of Korea

**Keywords:** older adults, dysphagia, swallowing, mHealth, thematic analysis, usability, apps, education, experience, sociodemographic

## Abstract

**Background:**

Swallowing difficulties (ie, dysphagia) are common among older adults, with a 13% to 54% prevalence. Adequate interventions to improve the swallowing function of older adults would reduce morbidity and enhance health-related quality of life outcomes. Mobile health (mHealth) apps may help alleviate dysphagia symptoms by providing programs that maximize the intensity and frequency of training without requiring high costs or regular clinic visits.

**Objective:**

The aim of this pilot study was to assess the usability of swallowing training apps by quantitatively and qualitatively evaluating older adults’ self-reported data, taking into consideration their educational levels and exposure to mobile technology. We conducted surveys and brief interviews while the participants used a swallowing intervention app we developed. We subsequently identified and resolved individual-specific usability issues to improve future implementation of the app protocol for older persons with swallowing difficulties.

**Methods:**

A total of 11 participants (10 women, 91%; mean age 75.7 years, SD 3.93) from two district-run senior welfare centers took part in this study. The participants were divided into a high-potential group and a low-potential group based on their total number of years of education and smart device usage. To investigate the usability of the app twice (ie, in the second week of the intervention and the postintervention stage), we used mixed methods consisting of both quantitative approaches, namely the System Usability Scale (SUS) and modified Computer Self-Efficacy Scale (mCSES) surveys, and qualitative approaches (ie, interviews).

**Results:**

The quantitative results of the SUS and mCSES surveys revealed that the high-potential group was more inclined to adopt and learn new technology than the low-potential group. Specifically, within the high-potential group, a Wilcoxon signed-rank test indicated that the postintervention mCSES scores (median 65.50) were significantly higher than those in the second week of intervention (median 54.00; *z*=–2.023, *P*=.04). Additionally, the usability scores in the low-potential group were within the “marginal acceptability” range even after completion of an 8-week intervention program. Qualitative analyses via semi-structured interviews yielded promising outcomes regarding app acceptability, training program utilization, emotional responses, and learning experience.

**Conclusions:**

To the best of the authors’ knowledge, this usability and feasibility study is the first report of a swallowing training app designed to improve the swallowing function of older adults. Future research should consider several issues, such as user characteristics, pretraining education, and the intensity and innate characteristics of the intervention program.

## Introduction

Condition-focused health issues, such as swallowing difficulties (ie, dysphagia), are prevalent among older adults. Approximately 13% to 54% of older adults are reported to have swallowing difficulties, depending on their age, underlying diseases, and care level [1-3]. In a study on the prevalence of swallowing difficulties among older Koreans, the age- and sex-standardized prevalence of dysphagia in Koreans aged 65 years or older was 98/415 participants (23.6%) [4]. Major complaints resulting from dysphagia include coughing or choking [2,5], spillage of food [5], and difficulty swallowing hard foods [6]. Because dysphagia in older adults increases their risk of malnutrition, dehydration, weight loss, and aspiration pneumonia [7], it is imperative to detect dysphagia symptoms early and provide adequate preventative efforts to reduce morbidity and improve health-related quality of life outcomes.

Rehabilitative interventions include swallowing-focused therapies such as the Mendelsohn maneuver [8,9] and effortful swallowing [10]. Indirect methods to improve swallowing also exist, such as the effortful pitch glide (EPG) exercise [11] and tongue rotation exercise [12]. These training exercises are usually conducted via interactions between swallowing clinicians and patients during therapy sessions at various health care facilities. However, to induce experience-dependent neural plasticity, repetition and intensity of training are key factors in motor behaviors [13] and swallowing regimes [14]. While goal-setting training usually requires regular clinic visits, a few studies [15,16] have reported methods of maximizing effective therapeutic parameters for better outcomes in terms of duration, frequency, and intensity of training.

However, older adults may experience barriers that make regular clinic visits a challenge. Several studies have reported that the following factors are associated with the difficulty faced by older adults when visiting clinics: expenses, distance, transportation, time, and greater difficulties associated with advanced age [17-19]. For example, one of the reasons for unmet health care needs among older Korean adults is economic adversity [20], which prevents these older adults from attending regular and intensive therapeutic training. Accordingly, mobile health (mHealth) technologies have tremendous potential to bring health care into the digital age by providing more efficient health care for people with limited resources—especially older adults, who may be less capable of visiting clinics on their own.

mHealth technology can be utilized to monitor, control, or deliver training exercises. Pervasive health monitoring or control via mHealth apps has been developed for various medical conditions, such as diabetes, hypertension, and headache [21,22]; some of these apps have proved to be successful [23]. However, self-exercises incorporating rehabilitation expand far beyond simply monitoring and controlling, and they may require a higher degree of patient adherence. It has been reported that nonadherence to home exercise in rehabilitation programs is as high as 50%; thus, more rigorous mHealth exercise systems that make use of coaching, self-monitoring, and education are needed to achieve the desired goals [24].

Some apps are commercially available for improving swallowing function; however, very few of these apps make use of swallowing research [25,26]. mHealth apps may benefit individuals who are capable of achieving the maximum intensity and frequency of training programs at home but cannot afford high health care costs. Through these apps, individuals may be able to alleviate their dysphagia symptoms. However, the use of mHealth apps to improve swallowing function is not commonly practiced among older adults. Although older adults can benefit from mHealth to address both general and specific health issues [27], they may encounter usability issues due to the complexity and unfamiliarity of the apps [28]. These issues may be more substantial among older adults with limited formal education and little previous experience of high technology. Thus, it is reasonable to presume that use of and receptivity to technology varies depending on these sociodemographic characteristics [29].

Thus, in this pilot study, we assessed the usability and feasibility of a swallowing training app by quantitatively and qualitatively evaluating self-reported data from older adults, taking into consideration their educational levels and exposure to mobile technology. Specifically, we conducted surveys and brief interviews while the study participants used a swallowing intervention app we developed. We thereby identified and resolved individual-specific usability and feasibility issues to improve future implementation of the app protocol for older adults with swallowing difficulties. In the current study, the term “usability” was defined as the degree to which the participants used the mHealth app as intended by the researchers, and “feasibility” was defined as the degree to which the app effectively assisted older adults in improving their swallowing difficulties [30].

## Methods

### Apps and Hardware

The 365 Healthy Swallowing Coach app was developed for the intervention program. The app was designed with two fundamental characteristics. First, it was simple to use irrespective of the user’s familiarity with mobile apps because the target app users were older adults. Second, it allowed users to effectively complete the swallowing exercises without a clinician physically present because older adults are generally unfamiliar with technology and often face physical and cognitive barriers [31].

**Figure 1:**
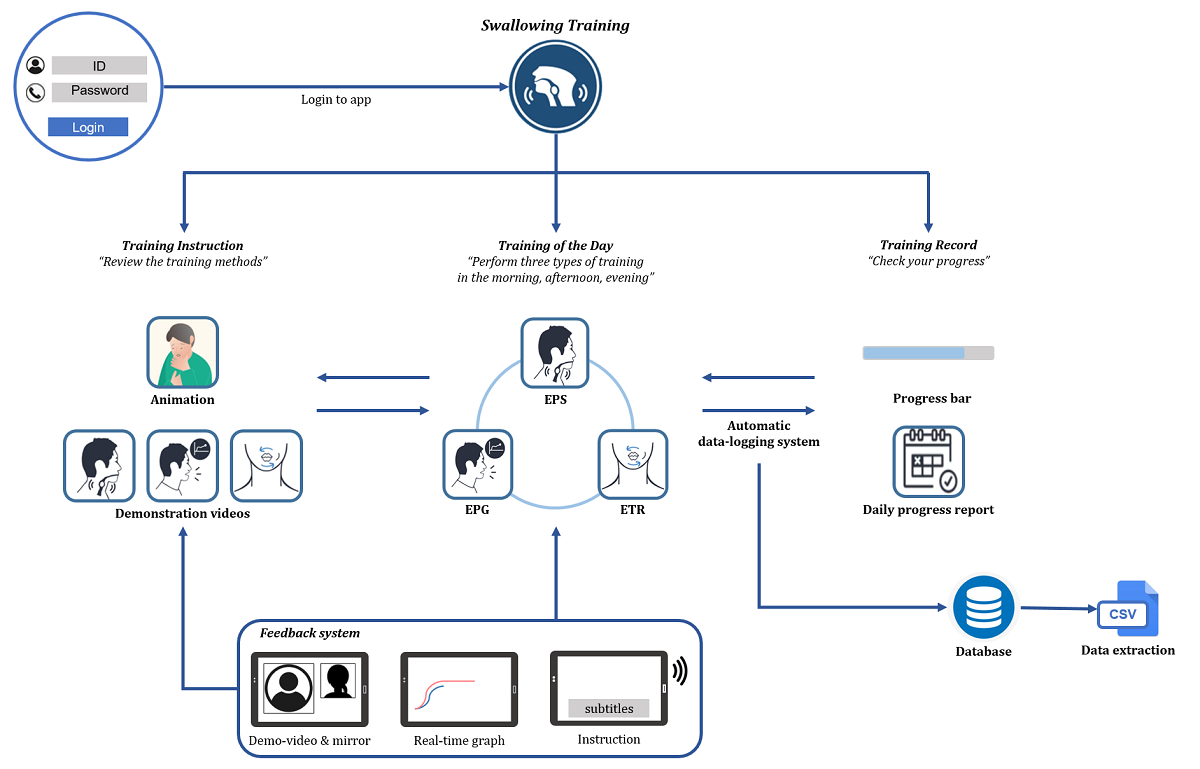
Overview of the 365 Healthy Swallowing Coach app. EPG: effortful pitch glide; EPS: effortful prolonged swallow; ETR: effortful tongue rotation.

We were particularly concerned with how well the users would navigate the app, reach the training page, and follow the training protocol. If the app required users to remember a complex sequence of actions, older adults might experience difficulties in navigating its functions [32]. Figure 1 provides an overview of the 365 Healthy Swallowing Coach app. In the first step, the user entered a user ID and password to log in. After logging in, the user was directed to the main page, Swallowing Training.

The Swallowing Training tab in the navigation menu enabled the users to access the Training Instruction, Training of the Day, and Training Record menus. The Training Instruction menu contained information regarding the training protocol, which was presented via animations and demonstration videos. Upon entering the Training of the Day menu, the user was shown three large tabs labeled Morning Training, Afternoon Training, and Evening Training. Each tab contained three exercises aimed at improving swallowing function: effortful prolonged swallow (EPS) [8-10], effortful pitch glide (EPG) [11], and effortful tongue rotation (ETR). [12] In our training protocol, users were required to complete 2 sets of 10 repetitions of each exercise in the morning, afternoon, and evening sessions (resulting in 60 repetitions of each exercise per day) on 5 days of the week of their choice. The app additionally included feedback system options, such as video demonstrations and a mirror function, real-time graphing, and audiovisual instructions, to monitor and correct the user’s performance [33]. 

**Figure 2:**
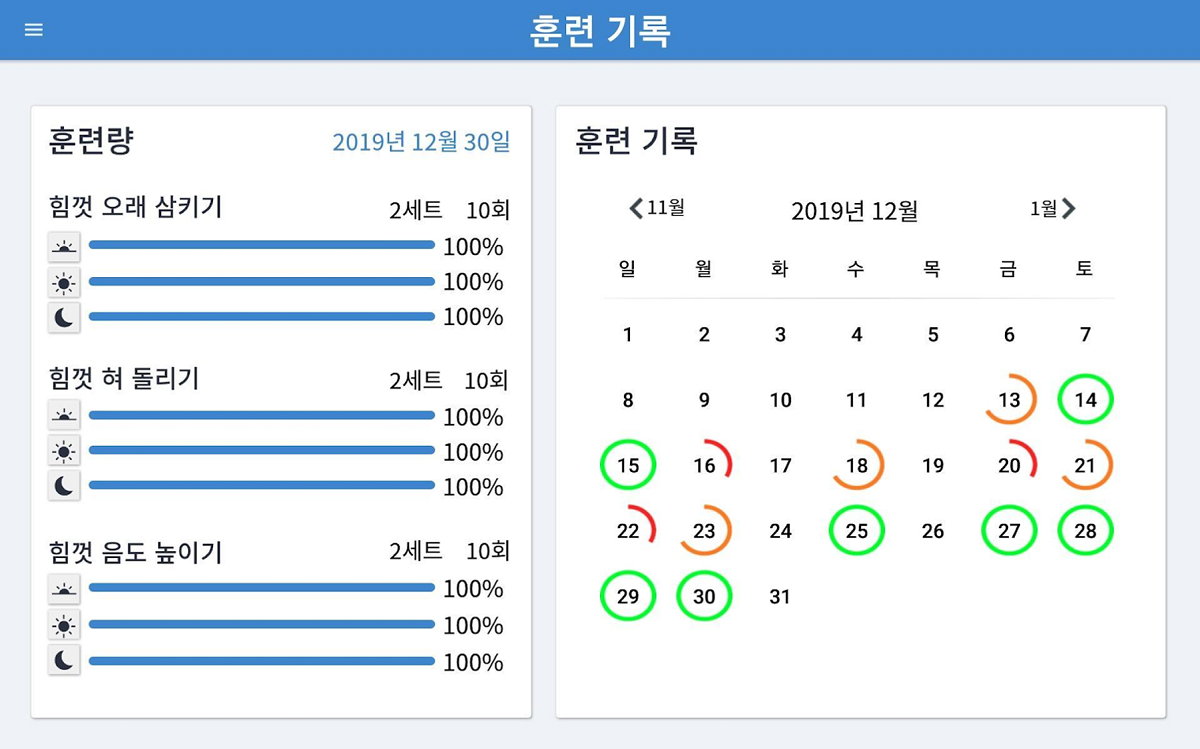
Screenshot of the Training Record screen of the 365 Healthy Swallowing Coach app.

The Training Record screen (Figure 2) enables users to keep track of the extent to which they have completed their exercises on any given day. On the left, the level of completion of each exercise in each session is indicated by bars and percentages; on the right is a calendar in which users can choose a day to review. The overall rate of completeness is marked on the calendar by a circle around each specific date. The Training of the Day screen also provides feedback on the user’s progress through a horizontal bar that “fills in” green as each training session is started and completed. When each training session is completed, the training data are automatically recorded through the automatic data-logging system and then saved in the database. The saved data can be extracted as comma-separated value (.csv) files when desired.

In addition, because limitations associated with aging may influence a user’s interactions with apps [34], the current app used a design that was specialized for older adults [35]. The buttons on the app contained both icons and text for easier viewing; these buttons were structured so that clicking anywhere on them performed the target action. In addition, to prevent users from pressing unwanted buttons by mistake, the sizes of the buttons and the spaces between them were adjusted appropriately and the color of the buttons was differentiated from that of the background. In particular, the buttons on the edges of the app were placed so that there was sufficient space between them and the soft buttons (ie, the Back and Recent Apps buttons) to prevent users from inadvertently pressing the latter. 

The prototype of the 365 Healthy Swallowing Coach app was specially designed to be compatible with a specific tablet PC model to minimize unforeseen bugs or errors after the launch of the intervention program. The app was built for the Galaxy Tab A model no. SM-P580 (Samsung Corporation) with a 1.6 GHz octa-core processor running the Android Oreo (version 8.1.0) operating system. The tablet has an internal storage capacity of 32 GB and 3 GB of RAM. The dimensions of the tablet are 254.3×164.2×8.2 millimeters, with a weight of 554 grams. The diagonal of the display dimensions is 255.4 mm, with a screen resolution of 1920×1200 pixels.

### Participants

The participants were recruited from two district-run welfare centers between November 11, 2019 and November 20, 2019. An advertisement for the study was placed in the centers. The individuals voluntarily presented themselves to research staff and were provided with a brief face-to-face introduction to the intervention program (eg, requirements for participation and training methods) by the authors HK, SHL, and NBC. This study was approved by the Institutional Review Board of the Yonsei University Health System (No. 4-2019-0888) and complied with the Helsinki Declaration of 1974 as revised in 2000. Written consent was obtained from all participants in the study.

Several inclusion criteria were established for the study. Participants were required to be older than 65 years; report swallowing difficulties (eg, experience aspirations more frequently than in the past or feel as if something is stuck in the throat); meet age and education level criteria for the Korean version of the Mini-Mental State Examination (K-MMSE) [36]; and have normal vision, hearing, and motor function of the upper limbs so they could use a tablet PC for the swallowing intervention. Specific exclusion criteria were also established. Individuals who had experienced neurological disorders and who required non-oral feeding (eg, nasogastric and percutaneous endoscopic gastrostomy tubes) were excluded.

### mHealth Intervention

**Figure 3:**
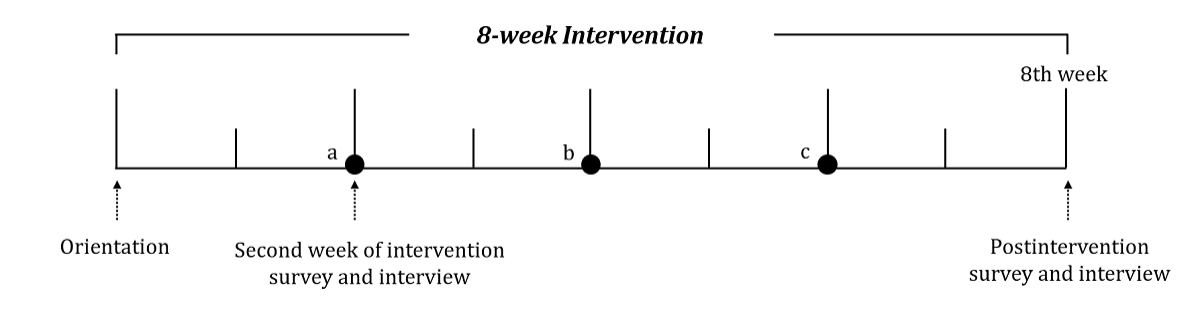
Timeline of the usability study. a,b,c: biweekly face-to-face meeting.

The swallowing intervention in this study consisted of an 8-week program (Figure 3). The participants were asked to engage in three different types of training (ie, EPS, EPG, and ETR) 20 times during a single session. The program consisted of 120 sessions (5 days a week, 3 times a day) in total. The participants also took part in four face-to-face meetings with the researchers. The first meeting (ie, orientation), which was held prior to commencement of the intervention, was designed to guide participants to learn how to use the 365 Healthy Swallowing Coach app and the tablet PC. Subsequently, meetings were held every other week to monitor the training progress and adherence of the participants to the program.

### Procedure

After written informed consent was obtained, demographic and socioeconomic status information was collected from the participants. Following this, they were asked if they had ever owned a mobile smart device, and if so, how long they had used or had been using it. Participants who reported having owned a mobile smart device were also asked if they had ever used a health-related app on their devices.

As shown in the usability study timeline (Figure 3), two short surveys were conducted in the second week of intervention and postintervention stages: the System Usability Scale (SUS) [37] and the modified Computer Self-Efficacy Scale (mCSES) [38], respectively. The SUS was conducted to gather information on the participants’ subjective judgments of the usability of the app, and the mCSES was conducted to evaluate their perceived confidence in coping with new technology. We translated the two scales into Korean with written email permission from the original authors for academic/nonprofit research use.

In addition, in the second week of the intervention and in the postintervention phase, semistructured interviews were conducted, and the results were used as the key source of information for our thematic analysis. The second week interview took place two weeks after the initiation of intervention program. The postintervention interview was conducted upon completion of the program. Both interviews were digitally recorded and transcribed verbatim. To thematically analyze the transcribed text, the participants’ statements were categorized into themes and subthemes depending on the context in which the responses were elicited.

### System Usability Scale

The SUS [37] is a widely used 10-item usability measurement scale. Several studies have demonstrated that the SUS is valid (ie, face, concurrent, and convergent validity) and reliable and that it can be used on a broad range of participants [39-41]. The scale was described by its creator as “a quick and dirty usability scale.” It is widely considered to be an effective usability assessment tool that can accommodate a variety of user interfaces [39]. The original SUS items are presented in Multimedia Appendix 1.

Responses to the SUS are rated on a 5-point scale from 1 to 5, with 1 corresponding to “strongly disagree” and 5 corresponding to “strongly agree.” The scoring system used to determine the overall SUS score is based on alternating positive items (nos. 1, 3, 5, 7, and 9) and negative items (nos. 2, 4, 6, 8, and 10). Responses to the odd-numbered items are subtracted by 1 and summed; responses to the even-numbered items are subtracted from 5 and summed. The sum of each subscore multiplied by 2.5 yields the total SUS score [42]. An example of the survey is presented in Multimedia Appendix 2.

### Modified Computer Self-Efficacy Scale

The original 10-item Computer Self-Efficacy Scale (CSES) was developed for workplace professionals by Compeau and Higgins [43] in 1995. Previous studies that used this or other questionnaires to assess perceived competence in using various types of technology have shown that higher self-efficacy is a crucial contributor to the likelihood of acceptance of the new technology. Laver et al [38] modified the original CSES (the mCSES) and tested its validity and reliability to determine whether it could be adapted to clinical rehabilitation settings for older and disabled populations. The results revealed high internal consistency, reliability, construct validity, and acceptance of the mCSES. The mCSES appeared to be suitable for our purpose of measuring the participants’ perceived confidence in coping with new technology. We made slight changes to wording of the mCSES to make it specific to mobile apps, such as changing the words “product” and “technology” to “app.” The mCSES items are presented in Multimedia Appendix 1.

The mCSES items are rated on a 10-point scale, ranging from 1 to 10, with 1 corresponding to “not confident at all” and 10 corresponding to “completely confident.” To calculate the total mCSES score, the point values of all 10 items are summed. The maximum score is 100 points [38].

### Interviews

The interviews were conducted by the authors HK, SHL, and NBC at 2 weeks and 8 weeks after the commencement of the intervention. At the time of the interviews, the first author (HK, female, PhD) was a university professor of a graduate program in speech-language pathology (SLP), with certificates in SLP (CCC-SLP, BC-ANCDS) and previous experience of conducting a qualitative study and a thematic analysis [44]. SHL and NBC (both male) were graduate students who conducted interviews under the supervision of the first author after interview training.

The items included in the first interview, which took place in the second week of the intervention program, were as follows: how adept participants believed they were at using the app; whether they experienced any difficulties when using the app, and how they coped with these issues; what participants’ opinions were regarding the strengths and weaknesses of the app; and whether the participants would like the app to be improved in any way. Depending on their responses, the participants were asked additional follow-up questions.

The second interview was conducted on the postintervention assessment day (ie, after 8 weeks of using the app). The second interview was identical to the first. The topic guide for the second week of the intervention and the postintervention is presented in Multimedia Appendix 3. The interviews were digitally recorded using an ICD-UX560F device (Sony Corporation) and transcribed verbatim for thematic analysis. The average duration of the interviews was 369 seconds (SD 141).

### Data Coding and Thematic Analysis

To identify key issues regarding the participants’ mHealth app usage, thematic analysis was used. We derived four major themes (ie, app acceptability, training program utilization, emotional responses, and learning experience) and multiple subthemes from the transcripts. The authors selected the aforementioned themes on the basis that the themes reflected patterns of responses in the interview data and were relevant to our research question [45], namely, “What factors affect the usability of swallowing training app for older adults?” The details of the themes are shown in Multimedia Appendix 4.

**Figure 4:**
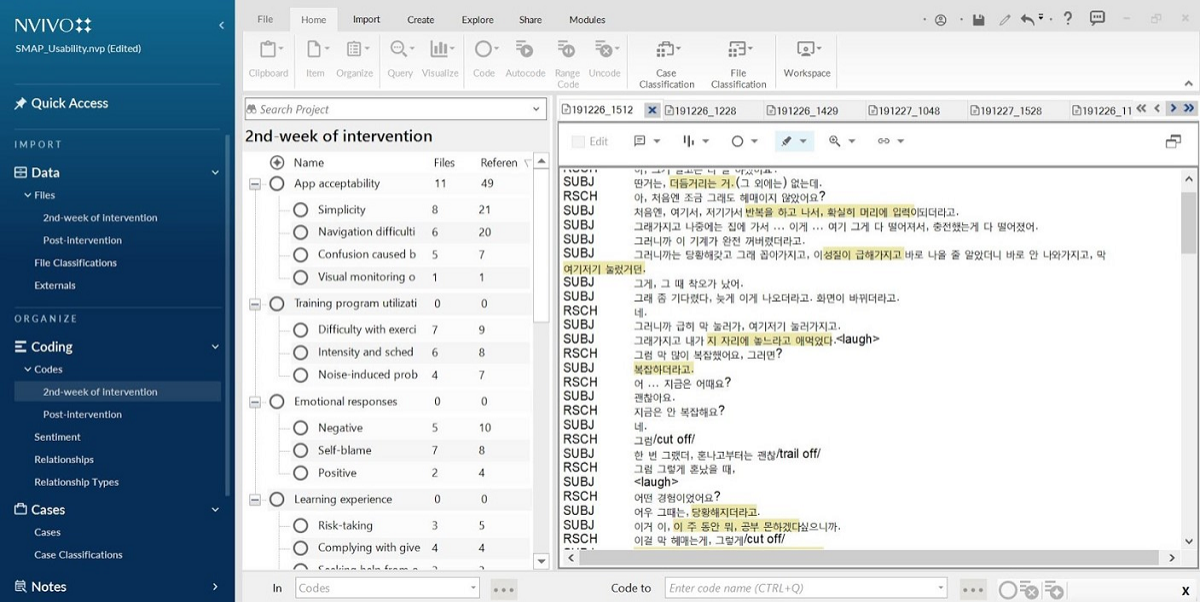
Example of the use of NVivo 1.0.

Data coding for the thematic analysis was conducted using NVivo software version 1.0 (QSR International) [46], which was used to code themes for the transcripts. Specifically, the first and second authors (HK and SHL) reviewed the interview data and selected specific phrases that were later assigned (using NVivo) to themes and subthemes according to their content. The selection of phrases and theme assignments were cross-checked by the three authors (HK, SHL, NBC). When disagreement occurred, the theme assigned by most of the authors was assigned to the utterance. The number of responses that corresponded to each theme was automatically calculated by the software. An example of the use of NVivo 1.0 is shown in Figure 4.

## Results

### Participants’ Characteristics

Eleven older adults participated in the study; 10 (91%) were female, and the mean age was 75.7 years (SD 3.93, range 67-83). This number met the optimal sample size requirement for a usability assessment [47]. All 11 participants successfully completed the 8-week intervention program. As shown in Table 1, the mean number of years of formal education was 9.9 (SD 3.36, range 5-16). The mean score of the K-MMSE was 28.2 (SD 1.26, range 24-30). Among the 11 participants, 8 (73%) reported currently owning or having previously owned a mobile smart device (ie, a smartphone), with the mean number of years of usage being 3.5 (SD 2.31, range 0-9). Among these 8 participants, only 2 (25%) had used a health-related app, such as a pedometer app (ie, a step-counter app), which can track a person’s number of steps and distance walked. The participants were divided into two groups based on their number of years of education and history of smart device usage (>10 years and ≤10 years, respectively): a high-potential group (n=6, mean age 74.3 years, SD 5.47; mean education 12.8 years, SD 3.00) and a low-potential group (n=5, mean age 77.4 years, SD 4.22; mean education 6.4 years, SD 1.52). There was statistical significance between the groups with regard to number of years of education (Mann-Whitney *u*=20.50, *P*=.004).

**Table 1:** Demographic characteristics of the participants (N=11).

Group and participants		Sex	Age (years; mean 75.7, SD 3.93)	Education (years; mean 9.9, SD 3.36)	K-MMSE score^a^ (points; mean 28.2, SD 1.26)	Smart device usage (years; mean 3.5, SD 2.31)	Health app use
**High-potential group (n=6)**							
	No. 1	F	77	12	27	3	No
	No. 2	F	73	12	28	9	No
	No. 3	F	83	9	24	5	No
	No. 4	F	71	16	29	2	Yes
	No. 5	M	75	16	28	8	Yes
	No. 6	F	67	12	30	4	No
	Mean (SD)	N/A^b^	74.3 (5.47)	12.8 (3.00)	27.7 (1.92)	5.2 (3.05)	N/A
**Low-potential group (n=5)**							
	No. 7	F	78	6	29	0	N/A
	No. 8	F	82	6	29	4	No
	No. 9	F	71	9	29	0	N/A
	No. 10	F	80	6	30	0	N/A
	No. 11	F	76	5	27	3	No
	Mean (SD)	N/A	77.4 (4.22)	6.4 (1.52)	28.8 (1.10)	1.4 (1.95)	N/A

^a^K-MMSE: Korean Mini-Mental State Examination, maximum score=30 points.

^b^N/A: not applicable.

### SUS Scores

As shown in Tables 2 and 3, within the high-potential group, a Wilcoxon signed-rank test indicated that the postintervention SUS scores (median 71.25) were not significantly different from scores obtained from the survey in the second week of intervention (median 73.75; *z*=–0.271, *P*=.79). Likewise, within the low-potential group, the postintervention SUS scores (median 70.00) were not significantly different from those in the second week of the intervention (median 55.00; *z*=–1.761, *P*=.08).

**Table 2:** Scores of the System Usability Scale (SUS) from the second-week and postintervention surveys for the high-potential and low-potential groups (N=11).

Group and survey		Mean (SD)	Minimum	Maximum	Median
**High-potential group (n=6)**					
	Second week of intervention	70.83 (8.75)	60.0	80.0	73.75
	Postintervention	72.08 (7.31)	65.0	82.5	71.25
**Low-potential group (n=5)**					
	Second week of intervention	56.00 (6.51)	50.0	65.0	55.00
	Postintervention	67.50 (7.28)	57.5	75.0	70.00

**Table 3:** Ranks and test statistics for the System Usability Scale (SUS) scores from the second-week and postintervention surveys for the high-potential and low-potential groups (N=11).

Group and rank		n	Rank (postintervention – second week)		Test statistics^a^ (postintervention – second week)	
			Mean rank	Sum of ranks	*z* value	*P* value
**High-potential group (n=6)**					–0.271^b^	.79
	Negative rank^c^	2	3.25	6.50		
	Positive rank^d^	3	2.83	8.50		
	Tied^e^	1	N/A^f^	N/A		
**Low-potential group (n=5)**					1.761^b^	.08
	Negative rank	1	1.00	1.00		
	Positive rank	4	3.50	14.00		
	Tied	0	N/A	N/A		

^a^Wilcoxon signed-rank test.

^b^Based on negative ranks.

^c^Postintervention score<score at second week of intervention.

^d^Postintervention score>score at second week of intervention.

^e^Postintervention score=score at second week of intervention.

^f^N/A: not applicable.

### mCSES Scores

As indicated in Tables 4 and 5, within the high-potential group, a Wilcoxon signed-rank test indicated that the postintervention mCSES scores (median 65.50) were significantly higher than those obtained during the second week of the intervention (median 54.00; *z*=–2.023, *P*=.04). In contrast, within the low-potential group, the postintervention mCSES scores (median 66.00) were not significantly different from those obtained during the second week of the intervention (median 48.00; *z*=–1.761, *P*=.08).

**Table 4:** Scores of the modified Computer Self-Efficacy Scale (mCSES) from the second-week and postintervention surveys for the high-potential and low-potential groups (N=11).

Group and survey		Mean (SD)	Minimum	Maximum	Median
**High-potential group (n=6)**					
	Second week of intervention	54.00 (17.28)	35.0	85.0	54.0
	Postintervention	65.00 (19.43)	38.0	96.0	65.5
**Low-potential group (n=5)**					
	Second week of intervention	44.44 (11.58)	29.0	57.0	48.0
	Postintervention	59.20 (14.92)	40.0	75.0	66.0

**Table 5:** Ranks and test statistics for the Computer Self-Efficacy Scale (mCSES) scores from the second-week and postintervention surveys for the high-potential and low-potential groups (N=11).

Group and rank		n	Rank (postintervention – second week)		Test statistics^a^ (postintervention – second week)		
			Mean rank	Sum of ranks		*z* value	*P* value
**High-potential group (n=6)**						–2.023^b^	.04
	Negative rank^c^	0	.00	.00			
	Positive rank^d^	5	3.00	15.00			
	Tied^e^	1	N/A^f^	N/A			
**Low-potential group (n=5)**						1.761^b^	.08
	Negative rank	1^c^	1.00	1.00			
	Positive rank	4^d^	3.50	14.00			
	Tied	0^e^	N/A	N/A			

^a^Wilcoxon signed-rank test.

^b^Based on negative ranks.

^c^Postintervention score<score at second week of intervention.

^d^Postintervention score>score at second week of intervention.

^e^Postintervention score=score at second week of intervention.

^f^N/A: not applicable.

### Thematic Analysis of Interview Content

As shown in Table 6 and Multimedia Appendix 5, we devised and defined themes to incorporate all content extracted from the interviews while minimizing the conceptual overlap among key themes. From the first and second interviews, we extracted 107 and 132 responses, respectively. Multimedia Appendix 5 (participants’ interview responses with 4 themes and 15 subthemes) presents the number of responses categorized within each theme and subtheme and the contrast in data between the high- and low-potential groups. We categorized the responses into four main themes; hereafter, the numbers in parentheses indicate the number of responses categorized into each theme in the first and second interviews, respectively. The themes selected were important app quality indicators according to the experiences of the participants: app acceptability (49; 51), training program utilization (24; 16), emotional responses (22; 37), and learning experience (12; 28). The theme of app acceptability, which pertained to different aspects of the app, was divided into four subthemes: simplicity (21; 17), navigation difficulties (20; 7), confusion caused by session selection (7; 17), and visual monitoring of exercise progress (1; 10).

Reports of navigation difficulties substantially decreased between the first and second interviews, possibly due to increased app experience. In contrast, the confusion caused by the session selection increased. This indicates that the design of the app and the location of the session selection buttons may have induced confusion among the older adult users when they selected the corresponding training sessions. Responses regarding the visual monitoring theme increased significantly between interviews, suggesting that the visual monitoring system of the app facilitated the self-monitoring process for the participants.

The theme of training program utilization encompassed the use of the training protocol and was divided into three subthemes: difficulty with exercises (9; 3), intensity and scheduling of the training protocol (8; 11), and noise-induced problems (7; 2). The participants reported experiencing less difficulty with exercises and noise-induced problems during the second interview than the first, which indicates that as the training program progressed, they were able to adapt to each of the training methods and cope with the noise-related issues mentioned during the first interview. In contrast, responses related to the intensity and scheduling of the training protocol were more frequent in the second interview. This suggests that the participants found that the intensity of the training protocol became more burdensome as they proceeded with the program.

The theme of emotional responses evoked by the use of the app was divided into three subthemes: negative responses (10; 9), self-blame (8; 17), and positive responses (4; 11). The incidence of self-blame and positive responses dramatically increased during the second interview, whereas the number of negative responses remained similar between interviews.

Finally, the theme of learning experiences was composed of five subthemes: risk-taking (5; 2), complying with given instructions (4; 5), seeking help from others (3; 5), progress in using the app (0; 12), and forgetfulness (0; 4). During the second interview, the users reported more incidences of complying with the given instructions and seeking help from others, while the incidence of risk-taking decreased. The three subthemes represent the way in which the older adults coped with issues regarding their experience with the app; the results suggest that the participants chose to adhere to the given instructions and ask for help instead of taking risks to solve problems as the program proceeded. The users also reported a significant increase in progress using the app, which indicates that the users were becoming skilled in app use. 

More responses were generated by members of the high-potential group (69; 78) than by members of the low-potential group (38; 54). In addition, the high-potential group reported better app acceptability in the second interview, as indicated by fewer navigation difficulties (13; 2) and greater perceived usefulness of visual monitoring of training progress (0; 9). In contrast, the low-potential group reported increased confusion caused by session selection, with an increase from 1 response in the first interview to 8 responses in the second interview. This suggests that at the time of the first interview, the low-potential group did not realize that they were not appropriately selecting the training sessions, and they only began to perceive the app as confusing once they realized that training session selection was something they needed to consider when following the exercise routines.

Regarding training program utilization, both groups reported less difficulty with exercises and fewer noise-induced problems in the second interview. In the high-potential group, the number of related responses decreased from 6 to 2, whereas in the low-potential group, the number of related responses decreased from 3 to 1. Interestingly, more high-potential participants reported the intensity and scheduling of the training program as being laborious in the second interview than in the first, as evidenced by an increase in the number of related comments from 4 to 11. Participants in the low-potential group expressed more self-blaming responses in the second interview than in the first (3; 9), although positive emotional responses also increased in frequency (2; 6). Finally, both groups demonstrated progress in app use competency after the 8-week intervention program.

**Table 6:** List of the 4 themes and 15 subthemes with examples of related statements.

Themes and subthemes		Example statements
**App acceptability**		
	Simplicity	*“Nothing was particularly complicated, everything showed up once I launched [the app].”*
	Navigation difficulties	*“I kept pushing this and that button but it still didn't work.”*
	Confusion caused by session selection	*“Even when I did all the sets, it doesn't say that I completed them. Why is that?”*
	Visual monitoring of exercise progress	*“Because of the green indicators, I was able to see what I missed.”*
**Training program utilization**		
	Difficulty with exercises	*“The one where you have to say 'eee' was the most difficult* *.* *”*
	Intensity and scheduling of the training protocol	*”Doing this three times a day is too much given my daily schedule.“*
	Noise-induced problems	*”I couldn't do [the pitch glide exercise] because I was concerned with alarming my neighbors.“*
**Emotional responses**		
	Negative	*”When [the app] didn't work as I wanted it to, I got annoyed.“*
	Self-blame	*”Old people like me need time to register things in the head.“*
	Positive	*”I was happy to learn something new.“*
**Learning experience**		
	Risk-taking	“*I touched the buttons here and there.”*
	Complying with given instructions	*”I was scared of getting lost, so I just stuck to what you taught me.“*
	Seeking help from others	*”I asked my grandson to help me.“*
	Progress in using the app	*”After figuring things out, it could not have been easier.“*
	Forgetfulness	*”When I go back home, I forget how.“*

## Discussion

### Principal Findings

mHealth is being increasingly recognized as an effective approach to deliver health care in a more accessible and cost-effective manner [48]. To the best of the authors’ knowledge, this usability and feasibility study is the first research on the use of swallowing training apps to improve the swallowing function of older adults. In this study, the home-based 365 Healthy Swallowing Coach app was used for 8 weeks by older adults. As a result, several issues emerged regarding the results of the analysis of the self-reported data.

First, user characteristics, such as the duration of formal education and smart device usage, were key variables related to usability, as reflected in the survey scores and interview data. Among the participants, we identified low-potential and high-potential groups based on their total years of education and smart device usage. In the second week of the intervention period, the low-potential group demonstrated a mean SUS score that fell within the “low marginal acceptability” range [39]. Even after completion of the training program, the mean SUS score of the low-potential group did not reach the “acceptable” category, falling within the “high marginal acceptability” range. In contrast, mean SUS score in the high-potential group fell within the “acceptable” range in both the second-week and postintervention surveys. Without previous experience using swallowing apps, the high-potential group reported the app to be promising in terms of feasibility and user satisfaction.

It was also interesting to note that regardless of their educational attainment and exposure to smart device usage, by the second week of the intervention program, both groups were unlikely to adopt a new app unless they were offered help and assistance with using it. However, as evidenced by the mCSES scores of the second survey, the high-potential group later gained confidence in using the new app; meanwhile, the low-potential group lacked confidence in adopting a new app even after completion of the 8-week intervention program. Thus, the discomfort with technology felt by older adults with diverse degrees of education and smart device usage should be approached differently to reduce socioeconomic disparity. For example, more extensive education or orientations regarding the use of the app would facilitate the execution of the intervention program among older adults, especially those with lower education levels [49]. A study has shown that even young older adults (ie, those over 50 years of age) had difficulties with certain aspects of smartphone usage after training, even though they demonstrated significant improvement in competency [50]. This suggests that there is a need for individually tailored education programs that consider user characteristics, such as smartphone proficiency and education.

Second, as both groups continued to learn via the app, the learning experiences accumulated and resulted in mixed learning outcomes. In this respect, we gained valuable insights from the qualitative data collected in the two interviews. For example, more participants claimed to have benefited from the self-monitoring features of the app in the second interview versus the first, which is consistent with previously suggested strategies for increasing adherence to home exercise [24]. In addition, the frequency of comments related to app navigation difficulties decreased. However, the frequency of self-directed blame for negative experiences with the app also increased, especially among individuals in the low-potential group. In previous research [51], “old age” or “aging” in older adults was cited as a main cause of functional limitation by 20% of the 230 participants. Identifying specific sources of aging-related issues in individual users, such as visual and hearing impairment as well as memory decline [18], would reduce self-blame and negative reactions to app use. Moreover, some older adults are passionate about acquiring new forms of technological skills that can aid them in maintaining their independence as well as their quality of life [29]. This is similar to a previous study in which it was reported that more than 80% of community-dwelling older adults expressed interest in using health technology [52].

Third, it is of the utmost importance to find the optimal balance among training intensity, frequency, duration, and level of adherence to maximize the benefits of app use among older adults. This balance is necessary because a discrepancy between the users’ perceived symptom severity and training intensity may harm their overall adherence [53]. Self-management and support while using apps without the presence of clinicians requires thorough scheduling and compliance with the schedule. Some participants in our study expressed dissatisfaction with the frequency of training; they claimed that three sessions a day (for a total of six sets of exercises per day) was excessive given that they had other plans or performed other activities during the day as well. Because patient compliance and adherence varies depending on the disease type and the participants’ perception of disease severity [53], the participants may have felt that the program was excessively time-consuming because they had a lower degree of swallowing difficulty and thus may have felt less motivation [54]. The optimal number (ie, frequency) and intensity of training sessions needed for older adults to induce experience-dependent neural plasticity [13,14] is a major concern when planning therapy programs. One study reported that the implementation of intervention programs in a “distributed” manner (6 hours/week, 8 weeks) resulted in significantly greater improvements compared to an “intensive” manner (16 hours per week, 3 weeks) for patients with aphasia [15]. The 365 Healthy Swallowing Coach app requires 7.5 hours per week, which is comparable to “distributed” therapy. Because the intensity needed for “intensive” swallowing exercise protocols varies [55], further research is needed to elucidate the relationship between the amount of training and therapeutic effects.

Fourth, the innate characteristics of an intervention program can create difficulties in its actual usage. For example, almost all of the reported difficulties in training program utilization referred to the effortful pitch glide (EPG) program, as high-pitched voice production during the program is a source of noise; this could be problematic, especially during the evening. Currently, no training technique has been established as an appropriate alternative to EPG. However, if there were options that could replace EPG, choosing the most appropriate option for home app use would be beneficial to increase the feasibility of the app. It is generally agreed that unlike treatment in clinical settings, which allow for patient-focused approaches, home therapies necessitate considering inspections of other complex factors, including the patient’s home environment and social context [56]. Therefore, until a substitute for EPG is found, a home environment assessment could be performed to determine whether to recommend a particular exercise.

### Limitations and Future Directions

Although it is the first usability and feasibility study of a swallowing training program for older adults, this study is not without limitations. First, all participants of this study are residents of Seoul, which is the largest metropolis in South Korea. Thus, further study is warranted to determine the extent to which the findings will be applicable to older adults from more rural regions.

Second, this study included only individuals who were 65 years of age or older (range 67-83 years). The inclusion of a broad age range of participants, such as the “oldest-old” (ie, people who are aged at least 85 years) [57], who are more susceptible to swallowing difficulties and have less experience with high technology, would reveal additional usability and feasibility issues with at-home training apps.

Further, this study only included participants with mild swallowing difficulties, which also limits the generalizability of the findings. Investigations of patients at increased risk or with a chronic course of dysphagia and with special intervention needs would fill major research gaps.

### Conclusions

Quantitative measurements may highlight the characteristics of different groups of users rather than indicate actual usability and feasibility problems. However, combined with qualitative data, we acquired insights into problems that older adults face when using mHealth technology as well as how a swallowing training app can be tailored to such persons. Qualitative investigation revealed that to provide an effective mHealth app to treat swallowing problems in older adults, the users’ years of formal education and smart device usage must be taken into consideration even during the training phase of app use. Additionally, despite some early difficulties as they became familiar with using the app, more participants expressed comfort with app usage later in the intervention, highlighting the potential of mHealth apps for older adults. However, self-blaming behavior may be a limitation when testing usability with older adults as evaluators. We also note the importance of our users’ home environment, intensity and adherence to the training program, and the nature of the training exercises in the usability and feasibility of our app.

## Appendix

Multimedia Appendix 1 System Usability Scale (SUS) and modified Computer Self-Efficacy Scale (mCSES).

Multimedia Appendix 2 An example of the System Usability Scale (SUS) response sheet.

Multimedia Appendix 3 Interview topic guide.

Multimedia Appendix 4 Codes for thematic analysis.

Multimedia Appendix 5 Participants’ interview responses with 4 themes and 15 subthemes.

## Abbreviations

CSES: computer self-efficacy scale
csv: comma-separated value
EPG: effortful pitch glide
EPS: effortful prolonged swallow
ETR: effortful tongue rotation
K-MMSE: Korean Mini-Mental State Examination
mCSES: modified Computer Self-Efficacy Scale
mHealth: mobile health
SLP: speech-language pathology
SUS: System Usability Scale
