# Supplementary material for: User-Dependent Usability and Feasibility of a Swallowing Training mHealth App for Older Adults: Mixed Methods Pilot Study
Source: JMIR Mhealth Uhealth. 2020 Jul 27;8(7):e19585. doi: 10.2196/19585 (PMC7418014; doi:10.2196/19585)
Supplement: Multimedia Appendix 1 [file mhealth_v8i7e19585_app1.pdf]

Multimedia Appendix. System Usability Scale (SUS) and modified Computer Self-Efficacy Scale (mCSES).

SUS

1. I think that I would like to use this system frequently.
2. I found the system unnecessarily complex.
3. I thought the system was easy to use.
4. I think that I would need the support of a technical person to be able to use this system.
5. I found the various functions in this system were well integrated.
6. I thought there was too much inconsistency in this system.
7. I would imagine that most people would learn to use this system very quickly.
8. I found the system very cumbersome to use.
9. I felt very confident using the system.
10. I needed to learn a lot of things before I could get going with this system.

Translated version of the SUS (Korean)

나는…

1. 이 애플리케이션을 자주 사용할 것 같다.
2. 이 애플리케이션이 불필요하게 복잡하다고 생각했다.
3. 이 애플리케이션이 사용하기 쉽다고 생각했다.
4. 이 애플리케이션을 사용하기 위해서는 전문가의 도움이 필요하다고 생각한다.
5. 이 애플리케이션의 다양한 기능들이 조화롭게 구성되어 있다고 생각했다.
6. 이 애플리케이션의 (만들어진 방식이) 일관성이 너무 없다고 생각했다.
7. 대부분의 사람들이 이 애플리케이션의 사용법을 빠르게 익힐 것이라고 생각한다.
8. 이 애플리케이션이 다루기 번거롭다고 생각했다.
9. 이 애플리케이션을 자신있게 사용했다.
10. 이 애플리케이션을 사용하기 위해 많은 것을 배우고 익혀야 했다.

mCSES

I could use the new technology ...

1. If there was no one around me to tell me what to do as I go.
2. If I had never used a product like it before.
3. If I had only the product manuals for reference.
4. If I had seen someone else using it before trying it myself.
5. If I could call someone for help if I got stuck.
6. If someone else had helped me get started.
7. If I had a lot of time to complete the job for which the product was provided.
8. If I had just the built-in help facility for assistance.
9. If someone showed me how to do it first.
10. If I had used similar products before this one to do the same job.

Translated version of the mCSES (Korean)

나는…

1. 내가 앱을 사용하고 있을 때 주변에서 어떻게 하라고 안한다면 이 새로운 앱을 사용할 수 있다.
2. 이와 비슷한 앱을 사용해본 적은 없지만 이 새로운 앱을 사용할 수 있다.
3. 설명서만 주어진다면 이 새로운 앱을 사용할 수 있다.
4. 내가 써보기 전에 다른 사람이 사용하는 것을 먼저 볼 수 있다면 이 새로운 앱을 사용할 수 있다.
5. 중간에 막힐 때에 도움을 청할 수 있다면 이 새로운 앱을 사용할 수 있다.
6. 시작할 때 누군가 도와주기만 한다면 이 새로운 앱을 사용할 수 있다.
7. 앱을 수행하는데 충분한 시간이 주어진다면 이 새로운 앱을 사용할 수 있다.
8. 앱 안에 도우미 기능이 있다면 이 새로운 앱을 사용할 수 있다.
9. 누군가가 나에게 먼저 어떻게 사용하는지를 보여준다면 이 새로운 앱을 사용할 수 있다.
10. 예전에 유사한 기능을 가진 앱을 사용한 적이 있었다면 이 새로운 앱을 사용할 수 있다.
