# Supplementary material for: User-Dependent Usability and Feasibility of a Swallowing Training mHealth App for Older Adults: Mixed Methods Pilot Study
Source: JMIR Mhealth Uhealth. 2020 Jul 27;8(7):e19585. doi: 10.2196/19585 (PMC7418014; doi:10.2196/19585)
Supplement: Multimedia Appendix 2 [file mhealth_v8i7e19585_app2.pdf]

Multimedia Appendix. An example of the System Usability Scale (SUS) response sheet.

## 사용성 평가 척도 (v.2)

각 문항을 잘 읽으시고, 1 점(매우 그렇지 않다) 부터 5 점(매우 그렇다) 중에 골라보세요.

나는...

**1. 이 애플리케이션을 자주 사용할 것 같다.**

| 매우 그렇지 않다 | 그렇지 않다 | 보통이다 | 그렇다 | 매우 그렇다 |
|---|---|---|---|---|
| 1 | 2 | 3 | 4 | 5 |

**2. 이 애플리케이션이 불필요하게 복잡하다고 생각했다.**

| 매우 그렇지 않다 | 그렇지 않다 | 보통이다 | 그렇다 | 매우 그렇다 |
|---|---|---|---|---|
| 1 | 2 | 3 | 4 | 5 |

**3. 이 애플리케이션이 사용하기 쉽다고 생각했다.**

| 매우 그렇지 않다 | 그렇지 않다 | 보통이다 | 그렇다 | 매우 그렇다 |
|---|---|---|---|---|
| 1 | 2 | 3 | 4 | 5 |

**4. 이 애플리케이션을 사용하기 위해서는 전문가의 도움이 필요하다고 생각한다.**

| 매우 그렇지 않다 | 그렇지 않다 | 보통이다 | 그렇다 | 매우 그렇다 |
|---|---|---|---|---|
| 1 | 2 | 3 | 4 | 5 |

**5. 이 애플리케이션의 다양한 기능들이 조화롭게 구성되어 있다고 생각했다.**

| 매우 그렇지 않다 | 그렇지 않다 | 보통이다 | 그렇다 | 매우 그렇다 |
|---|---|---|---|---|
| 1 | 2 | 3 | 4 | 5 |

**6. 이 애플리케이션의 (만들어진 방식이) 일관성이 너무 없다고 생각했다.**

| 매우 그렇지 않다 | 그렇지 않다 | 보통이다 | 그렇다 | 매우 그렇다 |
|---|---|---|---|---|
| 1 | 2 | 3 | 4 | 5 |

**7. 대부분의 사람들이 이 애플리케이션의 사용법을 빠르게 익힐 것이라고 생각한다.**

| 매우 그렇지 않다 | 그렇지 않다 | 보통이다 | 그렇다 | 매우 그렇다 |
|---|---|---|---|---|
| 1 | 2 | 3 | 4 | 5 |

**8. 이 애플리케이션이 다루기 번거롭다고 생각했다.**

| 매우 그렇지 않다 | 그렇지 않다 | 보통이다 | 그렇다 | 매우 그렇다 |
|---|---|---|---|---|
| 1 | 2 | 3 | 4 | 5 |

**9. 이 애플리케이션을 자신있게 사용했다.**

| 매우 그렇지 않다 | 그렇지 않다 | 보통이다 | 그렇다 | 매우 그렇다 |
|---|---|---|---|---|
| 1 | 2 | 3 | 4 | 5 |

10. 이 애플리케이션을 사용하기 위해 많은 것을 배우고 익혀야 했다.

| 매우 그렇지 않다 | 그렇지 않다 | 보통이다 | 그렇다 | 매우 그렇다 |
|---|---|---|---|---|
| 1 | 2 (circled) | 3 | 4 | 5 |

점수 산출 방법

① 각 홀수 문항(1, 3, 5, 7, 9)의 각 원점수에서 1 씩을 뺀다

② 5 에서 각 짝수 문항(2, 4, 6, 8, 10)의 각 원점수를 뺀다

③ 총점: 위의 (①+②)를 합한 값에 2.5 를 곱한다

계산식 (# 칸에 각 원점수를 넣음)

[(#-1)+(5-#)+(#-1)+(5-#)+(#-1)+(5-#)+(#-1)+(5-#)+(#-1)+(5-#)] × 2.5

=# × 2.5

=

대상자 점수

[(3-1)+(5-2)+(3-1)+(5-2)+(4-1)+(5-3)+(4-1)+(5-3)+(2-1)+(5-2)] × 2.5

= 24 × 2.5

= 60 (총점)
