# Supplementary material for: User-Dependent Usability and Feasibility of a Swallowing Training mHealth App for Older Adults: Mixed Methods Pilot Study
Source: JMIR Mhealth Uhealth. 2020 Jul 27;8(7):e19585. doi: 10.2196/19585 (PMC7418014; doi:10.2196/19585)
Supplement: Multimedia Appendix 3 [file mhealth_v8i7e19585_app3.pdf]

Multimedia Appendix: Interview topic guide.

[2nd-week of intervention]

1. You have used the swallowing training app for two weeks. How familiar are you with the app?

   - Probe: How long did it take for you to get familiar with the application or the training itself?

2. Did you feel that the app was simple enough to use without any help from the beginning? Or did you find it difficult to operate the app?

   - Probe: What process did you go through in order to become skillful at using the application?

   - If you found it difficult to operate the app in the beginning, what type of difficulties did you run into?

   - If you ran into any problems, what did you do to fix the problem?

3. Tell us what you think about the app in detail. What do you think are the pros and cons of the tablet-PC app?

   - Probe: Tell us what you thought was convenient or inconvenient while operating the app.

4. Should there be any improvements in the app itself?

   - Probe: Should there be improvements in regard to visual features/ settings/ trainings, etc.?

   - Did you find any errors while using the app?

5. Is there anything else that you would like to tell us about your experience with the app or the swallowing training?

[Post-intervention]

1. You have used the app for 8 weeks now. How familiar are you with the app now compared to when the last interview was conducted?

   - Probe: Do you have any new insights about the app that you didn't have previously?

   - What is your level of competence in using the app at the current moment?

2. Did you experience any additional troubles while using the app?

- Probe: If so, how did you deal with the problem?

3. If you have any new comments on the app, what is it in detail?

   - Probe: Did you find any other advantages or disadvantages regarding the app that you didn't find before? What was convenient and what wasn't?

   - Probe: Where the visual monitoring features/settings/training videos/ design of the app etc. helpful? Or did they induce confusion?

4. If you are to continue using the "365 Healthy Swallowing Coach," would you be able to use it without any problems?

   - If you were to use a new app that is familiar to this one (or a new device familiar to the current device), would you be able to use it without any difficulties?

5. Are there any additional comments about the app or the trainings in general?
