# Supplementary material for: User-Dependent Usability and Feasibility of a Swallowing Training mHealth App for Older Adults: Mixed Methods Pilot Study
Source: JMIR Mhealth Uhealth. 2020 Jul 27;8(7):e19585. doi: 10.2196/19585 (PMC7418014; doi:10.2196/19585)
Supplement: Multimedia Appendix 4 [file mhealth_v8i7e19585_app4.pdf]

Multimedia Appendix. Codes for thematic analysis.

(Sub)Theme	Description
App acceptability	Different aspects of the app which consists of simplicity, navigation difficulties, confusion caused by session selection, and visual monitoring of exercise progress
Simplicity	Simplicity of the app that aided the users' utilization of the app and contributed to positive experiences with the app
Navigation difficulties	Difficulty in navigating through the app to get to the wanted features such as demonstration videos and training sessions that hindered their use of and contributed to negative experiences with the app
Confusion caused by session selection	Confusion induced by the separate session selection tab (morning, afternoon, evening exercises) that hindered their use of and contributed to negative experiences with the app
Visual monitoring of exercise progress	Visual monitoring and feedback provided by the app that aided the users' utilization of the app and contributed to positive experiences with the app
Training program utilization	Responses related with utilizing the training program, which includes difficulty with exercises, intensity and scheduling of the training protocol, and noise-induced problems.
Difficulty with exercises	The difficulty level of the training protocols that hindered their use of the app and contributed to negative experiences with the app and the training program
Intensity and scheduling of the protocol	How the intensity and scheduling of the swallowing training protocol hindered their use of the app and contributed to negative experiences with the app

Noise-induced problem	The noise from the training demonstration or the level of noise that the app requires the users to produce (for the "Effortful Pitch Glide" exercise) that hindered their use of the app and contributed to negative experiences with the app and the training program
Emotional responses	Responses representing emotions of participants which includes negative, self-blame, and positive
Negative	Negative emotional responses evoked by the use of the app
Self-blame	Expressions indicative of the users' self-directed blame for negative experiences with the app
Positive	Positive emotional responses evoked by the use of the app
Learning experience	Learning experiences while using the app, which includes risk-taking, complying with given instructions, seeking help from others, making progress in using the app, and forgetfulness
Risk-taking	How the users took risks (e.g. pressing unknown buttons or going back to the home screen) to cope with the difficulties presented by usability issues or their own lack of understanding of the app
Complying with given instructions	How users complied to the given instructions to cope with the difficulties presented by usability issues or their own lack of understanding of the app
Seeking help from others	How the users sought help from others (e.g. family members or acquaintances) to cope with the difficulties presented by usability issues or their own lack of understanding of the app
Progress in using the app	The users' self-reported level of mastery in using the app and behaviours indicative of their mastery in using the app
Forgetfulness	The users' self-reported level of low mastery in using the app and behaviours indicative of their lack of ability in using the app due to forgetfulness
