# Supplementary material for: User-Dependent Usability and Feasibility of a Swallowing Training mHealth App for Older Adults: Mixed Methods Pilot Study
Source: JMIR Mhealth Uhealth. 2020 Jul 27;8(7):e19585. doi: 10.2196/19585 (PMC7418014; doi:10.2196/19585)
Supplement: Multimedia Appendix 5 [file mhealth_v8i7e19585_app5.pdf]

Multimedia Appendix. Participants' interview responses with 4 themes and 15 subthemes.

Themes	App acceptability (49; 51)				Training program utilization (24; 16)			Emotional responses (22; 37)		
Sub-themes	Simplicity	Navigation difficulties	Confusion caused by session selection	Visual Monitoring of exercise progress	Difficulty with exercises	Intensity & scheduling of protocol	Noise-induced problem	Negative	Self-blame	Positive
**Groups**										
Hp	15; 14	13; 2	6; 9	0; 9	6; 2	4; 11	6; 2	5; 5	5; 3	2; 5
Lp	6; 3	7; 5	1; 8	1; 1	3; 1	4; 0	1; 0	5; 4	3; 9	2; 6
Total	21; 17	20; 7	7; 17	1; 10	9; 3	8; 11	7; 2	10; 9	8; 17	4; 11

Themes	Learning experience (12; 28)					Total
Sub-themes	Risk-taking	Complying to given instructions	Seeking help from others	Progress in using the app	Forgetfulness	
**Groups**						
Hp	4; 1	2; 3	1; 3	0; 4	0; 0	69; 78
Lp	1; 1	2; 2	2; 2	0; 8	0; 4	38; 54
Total	5; 2	4; 5	3; 5	0; 12	0; 4	107; 132

Hp= high-potential group, Lp= low-potential group; In "$\#_1$; $\#_2$", $\#_1$= number of responses from the first interview (i.e., 2nd week of intervention interview), $\#_2$= number of responses from the second interview (i.e., post-intervention interview).
